# Pediatric Cranial Vault Lesions: A Tailored Approach According to Bony Involvement

**DOI:** 10.3390/children11111377

**Published:** 2024-11-13

**Authors:** Arianna Barbotti, Ignazio G. Vetrano, Cecilia Casali, Tommaso F. Galbiati, Sabrina Mariani, Edoardo Porto, Alessandra Erbetta, Stefano Chiaravalli, Laura G. Valentini

**Affiliations:** 1 Department of Neurosurgery, Fondazione IRCCS Istituto Neurologico Carlo Besta, 20133 Milan, Italy; arianna.barbotti@unimi.it (A.B.); cecilia.casali@istituto-besta.it (C.C.); tommaso.galbiati@istituto-besta.it (T.F.G.); sabrina.mariani@istituto-besta.it (S.M.); edoardo.porto@istituto-besta.it (E.P.); laura.valentini@istituto-besta.it (L.G.V.); 2 Department of Biomedical Sciences for Health, Università di Milano, 20122 Milan, Italy; 3 Department of Neuroradiology, Fondazione IRCCS Istituto Neurologico Carlo Besta, 20133 Milan, Italy; alessandra.erbetta@istituto-besta.it; 4 Pediatric Oncology, Fondazione IRCCS Istituto Nazionale dei Tumori, 20133 Milan, Italy; stefano.chiaravalli@istitutotumori.mi.it

**Keywords:** cranial vault, cranioplasty, dermoid, Langerhans cell histiocytosis

## Abstract

Background: Cranial vault lesions are common in children, with dermoid and epidermoid cysts being the most frequent. Management is debated due to their slow growth, but early resection can prevent complications and provide a definitive histological diagnosis, which is sometimes linked to systemic diseases. Methods: A retrospective study of children treated surgically for cranial vault tumors from January 2011 to April 2023 was conducted. The data collected included age, gender, symptoms, comorbidities, lesion location, radiological features, surgical techniques, histopathology, and recurrence rates. Results: Eighty-eight children (mean age: 3.5 years, mean tumor size: 1.21 cm) underwent surgery. The most common locations were the frontal and occipital bones. The main diagnoses were dermoid cysts, myofibroma, and Langerhans cell histiocytosis. Gross total resection was achieved in 64 cases with simple skin incisions. In 13 cases, small cranioplasties with bone cement were used. Craniotomy and cranioplasty with autologous bone grafting were performed for 11 patients with lesions larger than 2 cm and full skull thickness erosion. Conclusions: Early resection is recommended for complete removal with minimally invasive surgery and to ensure histological diagnosis. For lesions larger than 2 cm with full skull erosion, cranioplasty with autologous bone is the preferred technique.

## 1. Introduction

Cranial vault lesions can be diagnosed at any age, but dermoid and epidermoid cysts are among the most frequent skull lesions in the pediatric population [1,2,3]. These benign tumors are defined as ectodermal inclusion cysts; they originate from the entrapment of the surface ectoderm along the lines of skin fusion during embryologic development [3,4]. On the contrary, malignant lesions have an overall prevalence of 1 to 2%: rhabdomyosarcomas and melanocytic neuroectodermal tumors are known to occur in the scalp region [2,3]. The typical presentation is a palpable, painless lump [4,5,6]. Occasionally, they may cause pain on palpation; this characteristic could suggest a histological diagnosis different from dermoid and epidermoid cysts. Epidermoids and dermoids appear radiologically as small round or oval radiolucent lesions with a sclerotic margin [7]; malignant lesions with undefined margins with aggressive periosteal reactions cause bony destruction [7,8,9].

Considering their slow-growing and mainly benign behavior, the optimal management of typical cranial vault lesions in the pediatric population is debated, especially in solitary, unpainful masses [10]. Some authors suggest conservative management with regular follow-up [11,12]. On the other hand, early resection is recommended due to the potential adverse effects if these cysts are left untreated. Dermoids typically develop in areas where embryonic fusion occurs, such as the midline. The most common location for scalp dermoids is at the anterior fontanel, as this represents a significant point of midline fusion [4,13,14,15]. These midline cysts can extend intracranially or intradurally and tend to enlarge and erode the cranial bone [4,6,16,17]. Moreover, dermoid and epidermoid cysts undergo rupture or infection, also with intracranial involvement, potentially leading to abscesses and meningitis [16,18,19]. Surgical techniques significantly differ according to the presumptive diagnosis, based on clinical presentation and radiological features, the dimensions of the lesions, and the possible evolution [1,4,6,20,21]. Our study aims to assess the impact and role of various surgical techniques in a monocentric series of pediatric cranial vault tumors.

## 2. Materials and Methods

### 2.1. Patients’ Characteristics and Presurgical Evaluation

A longitudinal retrospective study was conducted on all consecutive children admitted to our institution for the surgical treatment of cranial vault tumors between January 2011 and April 2023. Data were retrieved from a prospectively collected database, including the age and gender of patients, clinical manifestations, comorbidities or congenital syndromes, location and number of lesions, radiological appearance, surgical techniques, histopathological diagnosis, and recurrence rate. Only subcutaneous lesions without bone involvement or erosion were excluded from the analysis. Along with physical examination and medical history evaluation, every child underwent ultrasonography, also with Doppler ultrasounds to analyze the vascular pattern and venous anomalies [12], and a CT scan with multiplanar 3D reconstruction to evaluate the presence and entity of bone invasion. In cases of a suspected diagnosis of malignancy or possible intracranial extension, a brain MRI was also performed.

### 2.2. Surgical Strategy and Postoperative Assessment

**Figure 1:**
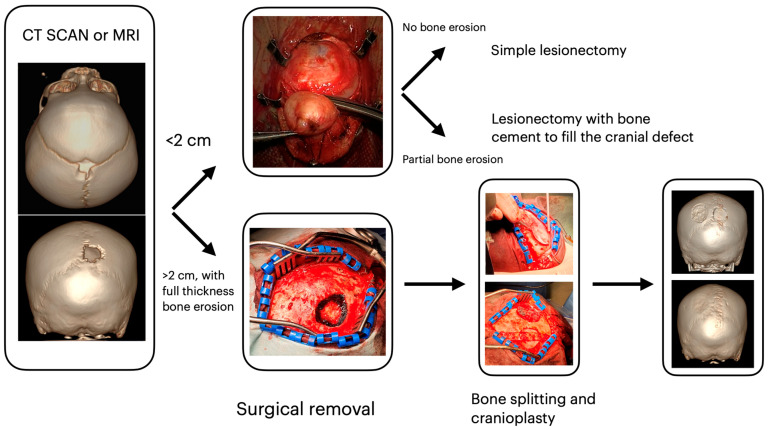
Surgical decision workflow according to lesion dimension and bone involvement.

A minimum size to perform a surgical treatment has not been considered as the cut-off to confirm or exclude surgical indications. Gross total en bloc resection was the aim of surgical treatment. The choice of the exact operative technique depended mainly on the cysts’ size and entity of bone invasion. For lesions smaller than 1 cm with partial thickness skull erosion, lesionectomy was performed: after a small skin incision just above the lump, the lesion is bluntly dissected free from the underlying calvarium using a dissector, avoiding cyst rupture that could lead to recurrence. For lesions between 1 and 3 cm, depending on the thickness of skull erosion, lesionectomy, as described above, with or without bone cement to fill cranial defects, is performed. Finally, craniotomy and cranioplasty, with autologous bone grafting, are conducted in case of lesions wider than 2 cm with full-thickness bone erosion (Figure 1). In case of epidural extension, depression, or thinning of the dura, a dural opening could be considered. Usually, all patients with small lesions who underwent minimally invasive surgery such as lesionectomy were discharged the same day or the following day after surgery. In cases of craniotomy, patients were discharged after 3 days. In the histologic diagnosis of dermoid and epidermoid cysts, patients had a clinical follow-up after 3 months and then annually. In the case of malignant tumors, children were assigned to the pediatric oncologist to receive adjuvant therapies. The subsequent follow-up was based on the specific oncologist’s indications. Surgical procedures, perioperative complications, and the need for second or further surgeries were also analyzed.

### 2.3. Statistical Analysis

Descriptive statistics were used to show the main sample features, specific numbers, and percentages for categorical variables, and means and standard deviation for quantitative variables. No statistical power calculation was conducted before the study; the sample size was based on the available data. All statistical analyses were performed using SPSS 24.0 statistical software.

## 3. Results

Between January 2011 and April 2023, 88 children underwent surgery for cranial vault tumors (Table 1). The mean age at presentation was 3 years and 6 months (SD ± 3.8), with a range from 5 months to 15 years and 9 months. There were 45 male patients and 43 female patients. In 73 cases, the sign leading to the clinical evaluation was a slow, progressively growing, painless palpable lump. On the other hand, in 13 cases, children reported local pain associated with rapidly enlarging masses (Table 1).

The distribution of the cranial vault tumors was as follows: frontal bone (n = 21), occipital bone (n = 17), bregmatic region (n = 9), periorbital region (n = 9), retroauricular (n = 7), temporal bone (n = 7), parietal bone (n = 7), pterional region (n = 5), glabella (n = 5), lambdoid suture (n = 1). Interestingly, in four cases of dermoid cysts, the children were also affected by craniosynostosis. The mean size of the lesions was 1,21 cm, with a wide range from 0.3 to 8 cm (SD 1.05).

### 3.1. Surgical Techniques

**Figure 2:**
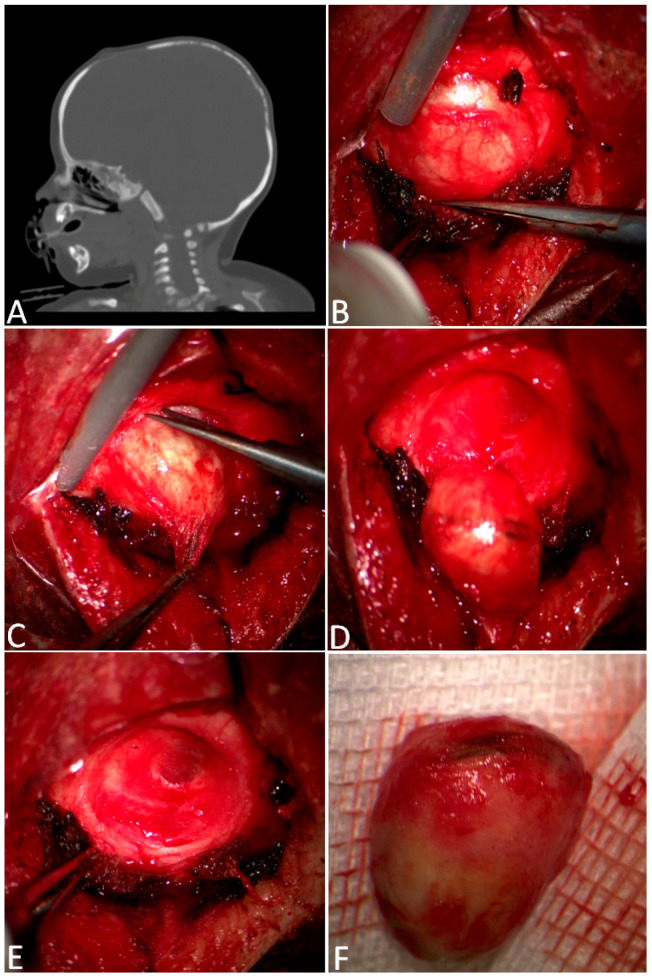
Complete removal of a frontal dermoid. In (**A**), the sagittal CT scans show a through-and-through bony defect slightly anterior to the bregmatic fontanel. After the skin incision, a subperiosteal lump was identified (**B**), represented by a well-encapsulated red–yellow mass eroding the whole thickness of the skull (**C**). The lesion, which was compatible with a dermoid, was enucleated (**D**). (**E**) shows an intraoperative view of frontal bone erosion after the en bloc removal of the dermoid cyst (**F**).

In 64 cases, a gross total resection was performed with a simple skin incision just above the cyst and enucleation of the lesion, followed by coagulation of the periosteal-free margins (Figure 2).

**Figure 3:**
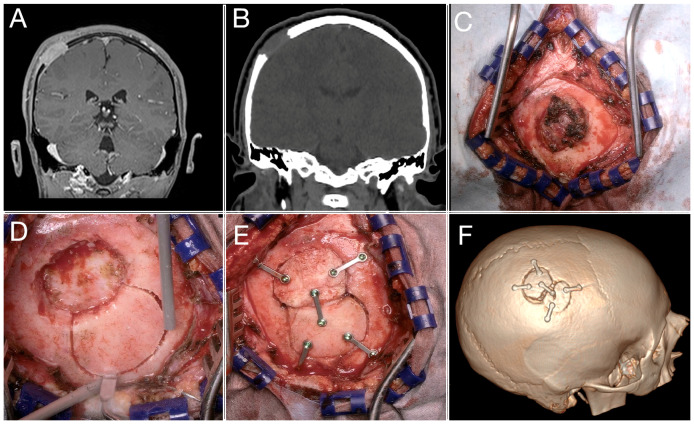
MRI and CT scans showing a parietal bone lesion extending in the epidural space (**A**,**B**); intraoperative appearance (**C**); bone-splitting procedure with piezo saw (**D**); final reconstruction (**E**); postoperative CT scan showing the closure of the bone defect (**F**).

In 13 cases, a small cranioplasty with bone cement was necessary to fill cranial defects, but always in older children (Figure 3). Finally, in 11 patients harboring lesions wider than 2 cm and full-thickness skull erosion, a craniotomy and a cranioplasty with autologous bone with split cranial bone grafting were performed. Considering the tripartite structure of the cranial vault (with a resilient inner and outer cortex separated by a diploic space), the presence in older children of soft cancellous bone within the diploe facilitates the bone splitting into separate fragments: an inner one and an outer one. In our series, the splitting technique was indicated in huge lesions, ranging from 2 to 8 cm, mainly in older children (mean age 8.3 years); in the majority of cases, the histopathological examination in such a specific cohort of children revealed Langerhans cell histiocytosis. The split bone was created close to the craniectomy performed for tumor removal; a fragment similar in size and shape was obtained and used as a graft for cranial vault reconstruction. In the case of dermoid cysts, the dural opening was never accomplished. Intradural exploration was conducted in just two patients undergoing intraoperative histopathological examination with the diagnosis of rhabdomyosarcoma and melanocytic neuroectodermal tumor.

### 3.2. Histopathological Examination and Follow-Up

The most frequent tumors were dermoid cysts (n = 62), followed by myofibroma (n = 9) and Langerhans cell histiocytosis (n = 9), and then cavernomas (n = 3), eosinophilic granuloma (n = 2), and others (1 osteoid osteoma, 1 rhabdomyosarcoma, 1 melanocytic neuroectodermal tumor). Among the 13 children with painful and rapidly growing lumps, 8 were diagnosed with histiocytosis, 2 with cavernoma, 2 with eosinophilic granuloma, and in 1 case, the histopathological diagnosis was rhabdomyosarcoma. In three Langerhans cell histiocytosis cases, the patients also presented associated fever. All children showed a regular postoperative course without complications, except for the child harboring a melanocytic neuroectodermal tumor, hence described. Among the patients with a histological diagnosis of Langerhans cell histiocytosis, in eight cases, no other extracranial localization was detected and patients had a radiological and clinical follow-up, as mentioned before; in one case, an extracranial localization was found and chemotherapy was started.

### 3.3. A Complex Case: Melanocytic Neuroectodermal Tumor

**Figure 4:**
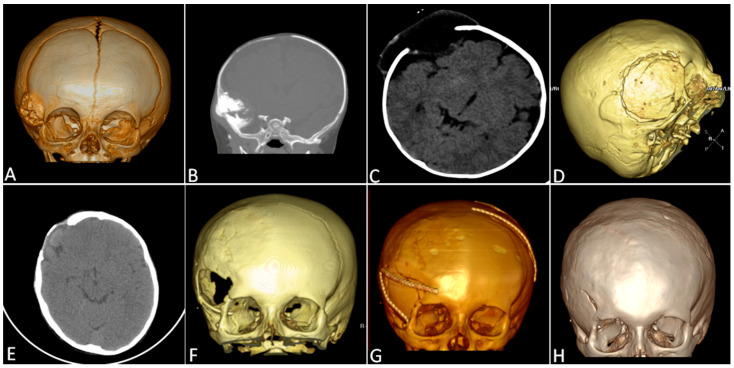
How to manage complications in wider tumors: a multistep approach. The three-dimensional CT scan depicts (**A**) a right frontotemporal intraosseous lesion, with intracranial extension ((**B**)—coronal CT scan). Surgery was performed, based only on tumor removal via craniectomy, without cranioplasty, to obtain re-ossification during growth; the histopathological diagnosis was of melanocytic neuroectodermal tumor. However, due to the onset of a large fluctuant lump (**C**), a second surgery with allograft bone was achieved (**D**): the CT shows the bone flap fixed with absorbable plates. After three years, central resorption of the bone graft was revealed (**E**), with progressive enlargement of the bone defect (**F**). Considering the currently sufficient thickness of the skull vault bone, the residual allograft was removed, and autologous bone grafting obtained with the “splitting” technique, secured with nonabsorbable plates, was performed (**G**). The last CT examination (**H**), 12 years after the first surgery, confirmed the absence of tumor recurrence and the complete re-ossification of the bone defect.

A 6-month-old child presented with a progressively growing, hard, and fixed mass in the right frontotemporal region for 4 months, before the parents sought medical attention (Figure 4). The CT scan showed a single hyperdense intraosseous lesion that appeared hyperintense on T1-weighted images and hypointense on T2-weighted images, with irregular contrast enhancement at MRI scans. Therefore, surgery was performed, based only on tumor removal via craniectomy, without performing cranioplasty. The postoperative course was uneventful, with a residual pterional bone defect, intending to obtain re-ossification during growth, thus avoiding a synthetic cranioplasty.

A few weeks after surgery, a large fluctuant lump was noted, diagnosed as pseudomeningocele at a new MRI. Due to its persistence despite punctures and compressive bandages, surgery with allograft bone was indicated after two months. The bone flap obtained from BioBank was shaped and fixed with absorbable plates. The child was then submitted to neuroradiological follow-up every six months. After three years, central resorption of the bone graft was revealed; the bone defect progressively increased over the next two years. Therefore, 6 years after the tumor removal, considering the sufficient thickness of the skull vault bone, we decided to remove the residual allograft and perform an autologous bone grafting obtained with the “splitting” technique, and then secured with nonabsorbable plates. Neither major complications nor tumor recurrence was observed, and the nonabsorbable plates were removed after two years. At the last follow-up, 147 months after surgery, no tumor recurrence was noted. The cranial bone flap showed full integrations within the craniectomy margins.

## 4. Discussion

Our retrospective study confirms that the surgical treatment of lesions of the skull vault in the pediatric population is safe and effective, with a very low rate of complications. Considering the fact that these cysts are mostly benign lesions, the optimal management in children is still debated. Specifically, major criticism arises over the correct management of a solitary, unpainful lump on a child’s head, knowing that the most frequent histopathological diagnosis is dermoid or epidermoid cyst. Some authors, mainly in the past, have suggested conservative management with planned follow-up [8,10,15]. On the other hand, early resection is recommended to prevent the potential adverse effects of leaving these cysts untreated. They tend to enlarge over time, causing the erosion of the cranial bone and potentially extending into the epidural space. This progression can lead to symptoms due to local mass effect, rupture, or even extension into surrounding structures, resulting in brain compression. In a study by Orozco-Covarrubias and colleagues, 66% of dermoid cysts increased in size, while 29% remained stable [22]. Also, Khalid et al. demonstrate that the delay in time to surgery significantly varies the degree of bone involvement [10]. These data are confirmed even in our series in which huge lesions, ranging from 2.3 to 8 cm and consequently needing cranioplasty, are found mainly in older children (mean age 8.4 years).

Furthermore, surgical resection gives the possibility of obtaining a histologic diagnosis to exclude malignancy. In our study, we did not find any correlation between patient age and malignant histology. However, we have observed—though this is based on clinical observation rather than statistical evidence—that lesions such as dermoid cysts, when left untreated, can fistulize and complicate the surgical procedure, which could make surgical excision more challenging. Also, cosmetic issues must be considered, and a shorter skin incision is possible for smaller cysts. As mentioned above, the goal of the surgical treatment was en bloc gross total resection. When performed on smaller cysts, complete and safe excision is generally easier to achieve, often without rupturing the cyst wall. All of these factors are positively associated with a reduced risk of recurrence [22]. As expected, dermoid cysts were the most frequent lesions in our series. Scalp dermoids are more frequent in areas of embryonic fusion such as the midline, or are related to the orbit and temporal bone sutures, considering that the inclusion of ectodermal tissue grows together with the membranous bones while a cranial suture is developing [4]. Our series confirms these findings: all nine lesions located at the bregma were dermoids, as were the majority of frontal lesions, along with all glabellar and lambdoid lesions (six in total).

As expected, in most cases, bone erosion did not involve the entire thickness of the bone, allowing for gross total resection through simple lesion enucleation. When infiltration was not present, the decision was made to preserve and close the periosteum to promote ossification. This technique avoids the need for allograft or synthetic composites, considering that resorption is the major risk for bone allograft. Such findings are also commonly reported in other large series. Prior et al., presenting a huge series of 234 pediatric dermoids and epidermoids, found that cranioplasty was never performed, considering both the reduced degree of skull erosion and the early diagnosis at a young age that allowed spontaneous bone repair during growth [6]. However, in their series, about 22% of the cysts eroded through a partial thickness of the cranium, 12% were in the entire thickness of the skull, and only 0.84% had epidural extension; therefore, two-thirds of cases showed non-significant or no skull involvement. Despite the consistent results in our series, particularly the favorable outcomes for small lesions in young children, we primarily focused on the variability of the surgical strategies used. All lesions in our series presented various cranial vault involvement and erosion, thus excluding pure subcutaneous lesions. The operative technique depended primarily on size and bone invasion. As mentioned above, according to our surgical workflow, lesions wider than 2 cm with full-thickness skull erosion need a cranioplasty to fill the bone gap. In the first years of life, the osteogenic potential of the dura allows smaller cranial defects to be left open; on the other hand, larger defects tend not to fully ossify, thus necessitating coverage with a graft [23]. In these cases, according to our experience, cranioplasty with split cranial bone grafting is the technique of choice. In fact, the cranium consists of a tripartite structure with a resilient inner and outer cortex separated by a diploic space. The diploic space is filled with soft cancellous bone that facilitates cranium splitting into separate bone fragments. Compared with bone from extracranial donor sites, autologous bone grafting is easily accessible, and it simulates more accurately the calvaria’s structure and function because it is harvested directly from the bone that it is replacing. Furthermore, it has been proven that autologous bone has the intrinsic property of promoting osteoconduction, osteoinduction, and osteogenesis and, consequently, expands with the rapidly growing brain [24].

Nevertheless, the thickness of the cranial vault changes between different ages of childhood; in infants less than 1 year, the splitting of the cranium into two separate bone fragments is not easy to conduct because the inner and the outer table of the cranium are more difficult to separate before the formation of the diploe. In situations where cranial bone graft is not available, another source of bone is the allograft. However, it must be underlined that the major risk for bone allograft is resorption, as our experience has shown: after three years from implantation, an area of resorption in the central portion of the bone flap was revealed upon radiological imaging, and the defect progressively increased over the next two years.

### Limitations

The main limitation of the current study is its retrospective nature. Nevertheless, we analyzed a monocentric homogeneous series with a standardized treatment approach and uniformity in the surgical team.

## 5. Conclusions

Dermoids are the most common childhood skull tumors. Despite their histological benignity, they tend to enlarge and erode the calvaria bone. Consequently, an early resection is recommended to obtain a complete and safe excision with minimally invasive surgery. In case of lesions wider than 2 cm with full-thickness skull erosion, a cranioplasty is necessary to fill the bone gap: cranioplasty with autologous bone with split cranial bone grafting is the technique of choice, according to the intrinsic property of cranial bone graft to promote osteoconduction, osteoinduction, and osteogenesis and to expand with the growing brain, with less risk of resorption than bone allografts. The same criteria were successfully applied in more rare skull lesions.

## Figures and Tables

**Table 1:** Summary of histological diagnoses in our cohort, including the number of patients who experienced pain and rapidly growing masses, as well as the number of patients undergoing adjuvant therapy.

Tumor Type	N°	Rapid Growth and Pain	Adjuvant Therapies
Dermoid cysts	62	0	0
Myofibroma	9	0	0
Langerhans cell histiocytosis	9	8	1
Cavernomas	3	2	0
Eosinophilic granuloma	2	2	1
Osteoid osteoma	1	0	0
Rhabdomyosarcoma	1	1	1
Melanocytic neuroectodermal tumor	1	1	1

## Data Availability

The datasets used and analyzed during the current study are available from the corresponding author on reasonable request.
